# Using Hot and Cool Measures to Phenotype and Predict Functional Outcomes Across Dimensions of ADHD and Typical Development in Adolescents

**DOI:** 10.1007/s10802-023-01149-7

**Published:** 2023-12-01

**Authors:** Heather Elahi, Ana-Maria Iosif, Prerona Mukherjee, Stephen P. Hinshaw, Julie B. Schweitzer

**Affiliations:** 1 Department of Psychiatry and Behavioral Sciences, University of California, Davis, Sacramento, CA 95817 USA; 2 MIND Institute, University of California, Davis, Sacramento, CA USA; 3 Department of Public Health Sciences, Division of Biostatistics, University of California, Davis, Davis, CA USA; 4 Department of Psychology, University of California, Berkeley, Berkeley, CA USA; 5 Department of Psychiatry and Behavioral Sciences, University of California, San Francisco, San Francisco, CA USA

**Keywords:** ADHD, Irritability, Emotional Lability, Cognitive Control, Impulsivity, Latent Profile Analysis, Adolescents

## Abstract

Multiple pathway models propose that attention deficit hyperactivity disorder (ADHD) arises from dysfunction in separate systems comprised of a "cool" or cognitive pathway versus a “hot” or emotional/reward pathway. Interactions between these pathways and the degree of maturation may further determine functional outcomes for adolescents ranging from those diagnosed with ADHD to typical development (TD). We used a latent profile analysis on rating scales and behavioral task performance assessing emotion, irritability, impulsivity, risk-taking, future orientation, and processing speed (PS) to identify subgroups of TD adolescents and adolescents with ADHD (*N* = 152) based on the hot and cool pathway model. We identified four classes: 1) *High-Complex Challenges*; 2) *Moderate-Mixed Challenges;* 3) *Non-Emotive Impulsivity*; and 4) *High Regulation and Control.* A multiple pathway model of ADHD is supported with classes differing in degree of emotional lability and irritability, types of impulsivity, and ability to use future consequences to modulate impulsivity and PS. The classes differed regarding functional behavior, with the *High-Complex* class demonstrating the most severe functional challenges in academic-related functioning. The *Moderate-Mixed* class also displayed significant functional challenges but with moderate emotional lability and irritability ratings. The *Non-Emotive Impulsivity* class exhibited low emotionality and low irritability, yet high impulsivity with limited negative functional consequences, and was composed of a mix of ADHD and TD adolescents. Differences between classes suggest ADHD symptomatology may represent both categorical and dimensional differences. Precision health interventions may be more effective in addressing the specific challenges associated with the classes rather than a one-size-fits-all approach to treating ADHD.

## Introduction

Attention deficit/hyperactivity disorder (ADHD) is one of the most diagnosed neurodevelopmental disorders, affecting 3–7% of US children aged 4 to 17 (Visser et al., 2014). The Diagnostic and Statistical Manual of Mental Disorders (DSM) classifies ADHD into three categories: predominately Inattentive presentation, predominantly Hyperactive/ Impulsive presentation, and Combined presentation (American Psychiatric Association, 2013). However, considerable inter-individual heterogeneity exists in the symptom presentation of individuals diagnosed with ADHD (American Psychiatric Association, 2013; Luo et al., 2019). Symptom-based subtypes have failed to demonstrate consistent external validity. They lack stability over time, have poor interrater reliability (Arnett & Flaherty, 2022), and lack distinct neuropsychological or neurobiological patterns (Willcutt et al., 2012). Parsing heterogeneity in ADHD to identify homogenous subtypes may advance specific guidelines for treatment options for the disorder (Arnett & Flaherty, 2022).

**Fig. 1:**
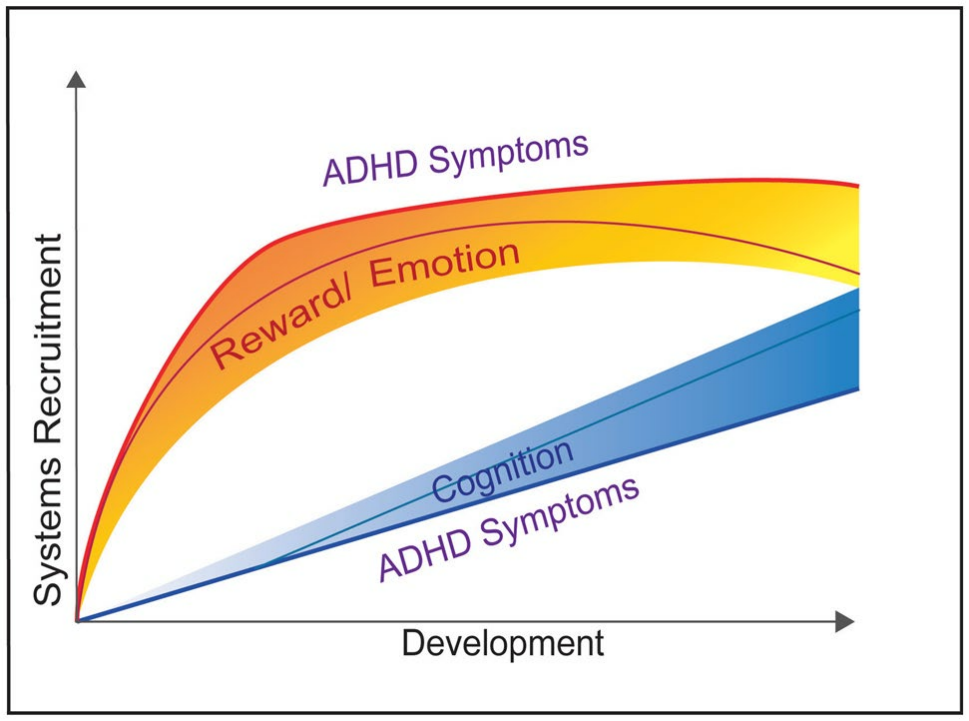
Dynamic model of ADHD Symptom Expression. Expression of ADHD symptoms is determined by the interactions between “hot” reward/emotion systems and “cool” cognitive control systems. Emotion/reward related processes peak earlier in adolescent development and remain steady for subgroups but decrease in other subgroups with development. Cognitive control processes increase linearly with development. Optimal regulation of emotion and behavior is achieved when cognitive control processes can effectively modulate reward/emotion processes either by reducing the pull of immediate rewards or enhancing the saliency of future, delayed rewards. Elevated ADHD symptoms may reflect either heightened reward/emotion systems, weak cognitive control systems, or a combination of the two. Elevated ADHD symptoms may also reflect emerging development with relatively immature cognitive control expected for one’s age. ADHD, attention-deficit/hyperactivity disorder

Researchers have attempted to conceptualize ADHD in various ways beyond the DSM categories (e.g., Nigg, 2017, 2022). For example, the Dual Pathway model in ADHD (Sonuga-Barke et al., 2010) emphasizes the contributions of cognitive control (“cool” processes) versus reward responsivity (“hot” processes) to explain individual variation in ADHD (Antonini et al., 2015; Castellanos et al., 2006; Skogli et al., 2017; Van Cauwenberge et al., 2015). In this model, ADHD symptoms arise from: 1) a deficit in cognitive control related to improper function of the dorsal lateral prefrontal cortex and/or; 2) an over-dependence on immediate rewards and mesolimbic dopamine dysfunction. The Triadic Model perspective for adolescent-motivated behavior (Ernst, 2014) also applies to ADHD during adolescence. It extends the Dual Pathway model by incorporating the critical role negative emotionality and irritability can have on reward-related processes (i.e., impulsivity and risk-taking) and higher-order cognitive operations. Furthermore, it incorporates the role development can play in how these systems interact with more advanced neurodevelopment associated with shifts in the balance toward higher future orientation and planned, thoughtful, cognitive control processes, away from automatic, visceral reward responding. Figure 1 presents a dynamic model of ADHD expression that considers the interaction between hot and cool processes and how their relation may change with development. The role of development is crucial to understanding ADHD, given that ADHD is associated with a delay in maturation by an average of 3 years in the cognitive control-brain systems (Shaw et al., 2007).

In the ADHD literature, cognitive control is often synonymous with the term “executive function” (EF) and used as an umbrella term referring to the cool cognitive processes necessary to complete goal-directed behaviors (Barkley, 2013). Children with ADHD display lower scores on cool EFs, including processing speed (PS) or how quickly an individual understands information and then acts on it (e.g., Chhabildas et al., 2001; Rucklidge & Tannock, 2002; Willcutt et al., 2005). Another cool EF is working memory (WM) performance, which involves holding information in mind and manipulating it to complete a task (Baddeley & Hitch, 1974), and it is well-documented as weaker in children with ADHD (Fassbender et al., 2011; Rapport et al., 2008; Ramos et al., 2020; Willcutt et al., 2005). On a neural level, studies have found that children with ADHD show reduced brain activation in several regions involved in WM, including the medial prefrontal cortex, the basal ganglia, and the cerebellum (Fassbender et al., 2011; Mukherjee et al., 2021). We note, however, that although WM difficulties are the most consistent cognitive impairment associated with ADHD, not all individuals with the diagnosis display challenges in WM (Kasper et al., 2012; Kofler et al., 2011; Nigg et al., 2018).

Hot EF challenges in ADHD, referring specifically to challenges in effectively managing emotions, are becoming increasingly studied and considered by some a key characteristic of the disorder (Barkley, 2015; Nigg, 2022). In addition, irritability, a narrower construct of emotional lability, defined by a short temper, low frustration tolerance, and sudden and unpredictable shifts toward negative emotions, is also progressively recognized as elevated in ADHD and associated with neural alterations (Kahle et al., 2021; Leibenluft et al., 2006; Maedgen & Carlson, 2000; Mukherjee et al., 2022; Nigg et al., 2004; Schweitzer el al., 2006).

Certain behaviors can be conceptualized as mediated by the interaction of cool and hot processes, such as risk-taking or situations involving deciding how to act toward a future reward or the absence of a reward (Lejuez et al., 2002). Weaker EF, stronger limbic-reward functioning, or reduced connectivity between EF-associated brain regions and limbic regions, as was found in typical development (e.g., amygdala, nucleus accumbens; van den Bos et al., 2015), may increase the likelihood of engaging in risky and impulsive behaviors. In everyday functioning, ADHD is associated with a plethora of risky behaviors such as higher rates of substance abuse, earlier pregnancies, lower likelihood to use protection during sex, more accidents, and emergency room visits compared to individuals without ADHD (Barkley et al., 2015).

Time perspective may mediate the balance between current behavior and future consequences and the interaction between cool and hot processes. Children with ADHD demonstrate poor time perception (Rubia et al., 2007). Weaknesses in future time orientation in ADHD have been hypothesized to be due to impairments in WM, inhibitory control, and present-time perception (Barkley, 2013). This may lead to a preference for present-oriented behaviors, such as engaging in impulsive actions, procrastinating, and avoiding tasks that have long-term benefits but require effort in the present (Barkley, 2013). Finally, understanding how cool and hot EF might relate to school performance and peer relationships is critical, given the frequent challenges in the school setting associated with the disorder (e.g., Polderman et al., 2010; Tamm et al., 2021).

### The Current Study

The present study aims to (a) use a person-centered approach to identify differential profiles of hot and cool processes and (b) characterize differences in sex, diagnosis, and performance among these profiles and relate them to areas of critical everyday functioning. We hypothesized that distinct profiles would emerge within the sample, beyond diagnostic categories—and that not all clinical participants would be rated as high on emotional variables. We then considered the relationship between profile membership and everyday functioning. This study focused on a sparsely studied group, adolescents with ADHD, Combined presentation symptoms, and TD adolescents, to enable the exploration of hot and cool processes at a time in development associated with rapid changes between these processes and in youth with heightened risk for impulsiveness and risk-taking behavior. We used latent profile analysis (LPA) to parse heterogeneity in ADHD and identify clinically meaningful subgroups. Furthermore, we applied the Research Domain Criteria framework to examine how phenotypic features associated with hot (e.g., emotional lability, irritability, risk-taking) and cool (e.g., PS, WM) functions presented beyond diagnostic categories in ADHD and TD youth, using a dimensional approach across a range of the hot versus cool measures.

## Methods

### Participants and Procedures

This study draws from the Mapping Impulsivity's Neurodevelopmental Trajectory (MINT) longitudinal investigation of the neurodevelopmental trajectory of impulsivity (PI: Schweitzer; Elliott et al., 2022; Kahle et al., 2021; Mukherjee et al., 2021, 2022) in adolescents and young adults. MINT collected imaging, behavioral, clinical, and academic measures for ADHD, Combined Presentation, and TD adolescents. Toward the end of the study, MINT also recruited participants with a range of ADHD symptoms between TD and the criterion for clinical diagnosis of ADHD to study ADHD symptoms on a continuum. The University of California, Davis Institutional Review Board approved this study, with written informed consent obtained from parents and written assent from participants.

Participants were recruited from the MIND Institute participant recruitment pool, MIND Institute and Departments of Psychiatry and Pediatric specialty clinics, local middle and high schools and universities, community flyers, and social media sites. Two licensed psychologists (JBS and JFD) evaluated phone screen data regarding symptoms and functioning to determine study eligibility. Eligible participants and one of their parents/caregivers completed a full psychiatric interview (Diagnostic Interview Schedule for Child and Adolescents or the M.I.N.I. International Neuropsychiatric Interview—Kid for DSM-5); parents and teachers completed rating scales (i.e., Parent [Conner-3 Parent Rating Scale—CPRS-3]) and [Teacher (Conners-3 Teacher Rating Scale—CTRS-3]). A licensed psychologist (JBS or JFD) determined whether participants met DSM criteria for ADHD, the presence of any other major psychiatric disorder, or would be best classified as “subthreshold” or TD. Subthreshold ADHD refers to a classification in which individuals exhibit the presence of symptoms characteristic of ADHD but do not meet the full diagnostic criteria as outlined in the DSM-5. Specifically, individuals categorized as subthreshold ADHD in our study present with 3–5 symptoms related to either inattentive or hyperactive/impulsive behaviors, which are representative of ADHD symptomatology. Moreover, these symptoms are accompanied by substantial functional impairment across multiple settings, indicating their significant impact on daily activities, responsibilities, and interactions.

Initially, DSM-IV-TR was used, and then DSM-5 upon its publication. However, evaluation criteria for ADHD for participants entering the study under DSM-IV-TR were re-reviewed for DSM-5 criteria for ADHD for all included participants. Inclusion criteria included an IQ ≥ 80 with additional inclusion criteria for the ADHD group meeting DSM-5 criteria for ADHD Combined presentation. Exclusion criteria included the presence of a math or reading learning disability; a history of head trauma, neurological disorder, or major medical problem as reported by the participant or their parents; taking psychoactive medication other than stimulants or atomoxetine; and meeting criteria for Axis I diagnosis except for ADHD, oppositional defiant disorder, or conduct disorder. Participants prescribed stimulant medication or atomoxetine for ADHD abstained from taking the medication for five half-lives before being tested on the Balloon Analogue Risk Task and Picture Order Memory Paradigm. The current study focuses on the adolescents in our study and includes 152 youth between 12 and 17.9 years old (99 boys, 53 girls), utilizing the data from their baseline visit.

### Measures

We selected a range of measures that tap into cognitive control and reward/emotion processes and functions that integrate the two. We used multi-informant reporters, including parent and self-report scales and objective measures, representing trait and state functioning.

#### Conners’ Parent Rating Scale 3rd Edition

We used the long-form CPRS-3 to evaluate ADHD and associated symptoms, including the DSM Hyperactivity/Impulsivity, Emotional Lability, EF, Learning Problems, and Peer Relations scales. We also derived an Irritability measure based on summing five items (i.e., 12, 48, 73, 81, 100) from the CPRS-3 shown to highly correspond to items on the Affective Reactivity Index (Kahle et al., 2021; Stringaris et al., 2012). There is no overlap in the items included in the Emotional Lability and Irritability scales.

#### Barratt Impulsiveness Scale (BIS-11)

This scale is the most widely cited questionnaire designed to assess the behavioral construct of impulsiveness. It consists of 30 items based on a self-report questionnaire. We used the Motor Impulsiveness and Self-Control factors of the BIS, as these two are most consistent with our goal of studying impulsivity. The Motor Impulsiveness factor captures challenges in inhibiting acting, whereas the Self-Control factor refers more to problems with delaying gratification. Therefore, the present study extracted motor impulsiveness and self-control raw scores to use in the LPA.

#### Balloon Analogue Risk Task (BART)

The BART is a computerized decision-making task used to assess risk-taking behavior (Barnhart & Beulow, 2017). The BART has good internal consistency, test–retest reliability, and convergent validity (Lejuez et al., 2002). It simulates real-world situations involving risky behavior, where more balloon pumps can yield more money. However, higher pumps can result in the balloon popping, with a loss of all the money. Participants were given real money based on their performance, and the average amount earned from the BART was used in the LPA.

#### Wechsler Scales of Intelligence

Participants aged 12—16 years completed the full Wechsler Scale of Intelligence (WISC-IV) to assess intellectual ability; participants above 16 took the Wechsler Adult Scale of Intelligence (WAIS). We utilized the PS composite score for the LPA, composed of two subtests: coding and symbol search.

#### Zimbardo Time Perspective Inventory (ZTPI)

The ZTPI is a valid and reliable index of individual differences in time perspective (Sircova et al., 2014; Zimbardo & Boyd, 1999). Time perspective corresponds to an individual’s view of the past and future at a given time. The present study uses raw scores from present hedonism and future orientation in the LPA. Present hedonism refers to being drawn to a reward and satisfying oneself at the time. Future orientation refers to using future consequences to guide current behavior and involves the ability to consider the future and planning. Prior findings from our group demonstrated adolescents had lower future orientation scores than young adults (van den Bos et al., 2015), and ADHD had lower future orientation and higher present hedonism than TD youth (Elliott et al., 2022). The ZTPI was added partway through data collection; thus, we substituted data from the next available visit for participants missing this measure at baseline.

#### Wechsler Individual Achievement Test (WIAT-III)

The WIAT–3rd Edition is an individually administered, norm-referenced test evaluating a range of academic skills across several domains. In the current study, we aimed to assess differences in functional outcomes of reading comprehension and math problem-solving among LPA classes.

#### Picture Order Memory Paradigm (POMP)

The POMP (Mukherjee et al., 2021) requires WM using a condition in which participants are shown three images sequentially, followed by instructions to recall the items in reverse order (i.e., backward) (Mukherjee et al., 2021). We used the total number correct on the task for the analysis. Our earlier work demonstrated neural and behavioral differences between TD and ADHD groups (Mukherjee et al., 2021). In this study, we assessed how the functional outcome of this WM task differs across the LPA classes.

#### Relation Between Classes and Broader Functional Outcomes

We used broad, day-to-day functioning measures to explore their relation with the identified classes. Measures of broader functioning included those mentioned above from the CPRS-3: ADHD Predominantly Hyperactive/Impulsive DSM-5 symptom scale, Peer Relations, Learning Problems, and Executive Functioning subscales. Additionally, the WIAT-III assessed Reading Comprehension and Math Problem-Solving abilities. Finally, we evaluated the relation between the classes and a measure of WM (POMP), requiring strong manipulation of information given that WM issues are prevalent in ADHD and hypothesized to underlie many critical EF processes in ADHD (Rapport et al., 2008).

### Statistical Approach

We first used LPA to identify distinct patterns related to emotional and reward functioning and cognitive control based on CPRS-3, BIS, ZTPI, and BART scores. The maximal reliability (*H*) for the eight variables included in the LPA was 0.92 (95% confidence interval 0.88 to 0.95). Models were estimated using full-information maximum likelihood, allowing us to include the participants with missing data under the missing-at-random assumption. We fitted and compared models with increasing numbers of classes, starting with one and determining the optimal number of classes based on statistical goodness-of-fit criteria, considering whether the classes capture clinically meaningful features and the proportion of participants represented in the classes (Masyn, 2013; Nylund et al., 2007). Goodness-of-fit criteria included Bayesian information criterion (BIC) and sample-size adjusted BIC, Akaike Information Criterion (AIC), consistent AIC and corrected AIC, approximate weight of evidence criterion (AWE, Masyn, 2013), correct model probability (cmP), Vu-Lo-Mendell-Rubin, Lo-Mendell-Rubin adjusted (LMR), and Parametric Bootstrapped likelihood ratio tests (BLRT, Lo et al., 2001; Nylund et al., 2007). Smaller AIC, BIC, and AWE values indicate better fit. cmP allows a researcher to compare a set of more than two latent class models, and higher values indicate more robust evidence for the candidate model compared to other models (Masyn, 2013). The likelihood ratio tests compare the fit of the specified class solution to models with one fewer class, and a significant *p*-value indicates that the specified model is preferred. The local maximum problem was addressed using up to 2,000 starting points to replicate each model.

Each LPA model identifies the number of latent classes (subgroups) within the sample and estimates posterior probabilities for each participant’s assignment to each latent class. For descriptive analyses, the highest posterior probability from the best-fitting model was used to assign each participant to the most likely subgroup. For subsequent analyses using latent subgroup membership (i.e., examination of differences in diagnosis, sex, impulsivity, and achievement), multiple pseudo-class draws (Bandeen-Roche et al., 1997) were used to reduce bias by accounting for the uncertainty in class assignments. Differences in categorical characteristics (e.g., sex, ADHD diagnosis) across latent classes were assessed using *χ*^*2*^ tests. Differences in dimensional measures of functioning were assessed using general linear models, accounting for age and sex, as appropriate. Age and sex were not retained in the reported models if they did not contribute significantly to the model. Transformations were employed for variables that violated the normality assumption. We used univariate and bivariate residual plots and univariate summaries of residuals to check the normality and homoscedasticity of residuals. Participants were randomly classified into latent classes 100 times based on their distribution of posterior probabilities from the best-fitting LPA model. The subsequent analyses were performed 100 times (i.e., for each draw), and results were combined across draws using standard methods for multiple imputations for missing data (Rubin, 1987). After combining results across draws, two approaches were used to control for multiple comparisons when assessing significant differences between groups. First, overall, three-degree-of-freedom *F*-tests for the group were adjusted for multiple comparisons using the Benjamini–Hochberg method. Then, for all variables with significant overall *F*-tests for group after adjustment, a second Benjamini–Hochberg procedure was used to identify all pairs of latent groups that were significantly different out of all possible pairs. LPA was performed in *Mplus* version 8.0 (Muthen & Muthén, 1998-2017). All other analyses were implemented using SAS Version 9.4 (SAS Institute Inc., Cary, NC). All tests were two-sided, and *p*-values < 0.05 were considered statistically significant.

## Results

The analyzed sample consisted of all study participants who were less than 18 years old at their baseline visit and included 152 youth (53 females, 99 males), of which *n* = 83 (24 females, 59 males) had ADHD, *n* = 6 (3 females, 3 males) had subthreshold ADHD, and *n* = 63 (26 females, 37 males) were TD. Of those reporting race, the sample was predominantly (90.7%) White, 19.7% Hispanic, and 78.8% had at least one parent with a college or more advanced degree.

### Latent Profile Analyses

**Fig. 2:**
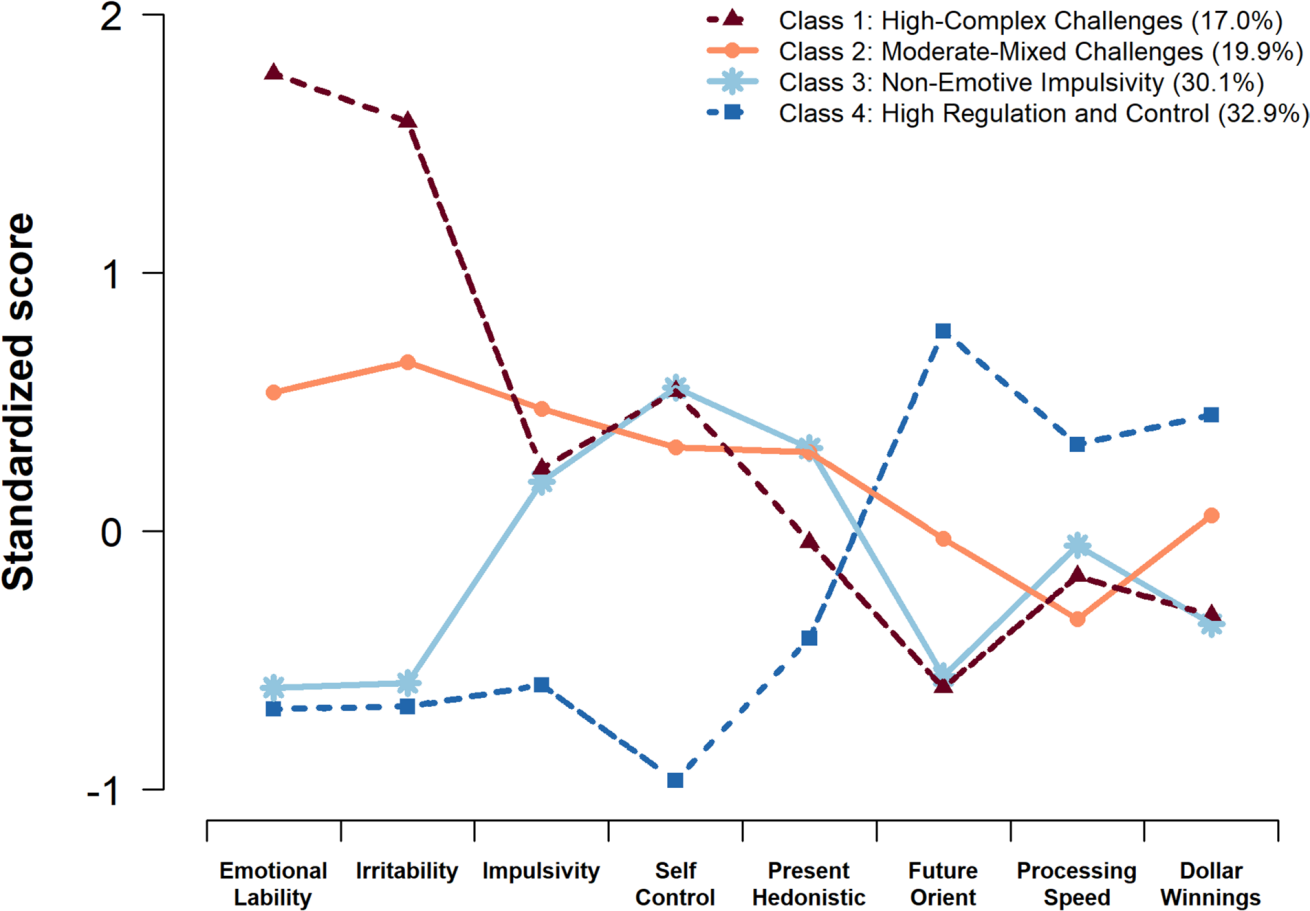
Profiles of the four LPA classes. To help the interpretation of this graph, variables were standardized to a mean of 0 and a standard deviation of 1

Fit indices for one-class to six-class solutions are summarized in Table 1. They provided support for the four-class as the optimal solution. Except for AIC and sample-size adjusted BIC indices, which never increased with added classes (though substantially smaller improvements were garnered after the four-class solution), the AWE, which supported a two-class solution, and the BLRT, which continued to support the larger model up to six classes, all the other criteria suggested that a four-class solution was optimal (four-class was better than three-class, and five-class was not better than four-class). In latent profile analyses, AIC and BIC may not increase with additional parameters, but the resulting models may have additional classes that are not meaningful. For example, in the five-class model, one class with impairments across modalities was differentiated into two classes that were not meaningfully different. Moreover, the five- and six-class models identified a class that included 5% or less of the sample. Thus, the four-class solution was selected as the most parsimonious model that still provided adequate fit and the most clinically meaningful distribution of classes. Based on the pattern of profiles, the four classes were named *High-Complex Challenges* (17.0%), *Moderate-Mixed Challenges* (19.9%), *Non-Emotive Impulsivity* (30.1%), and *High Regulation and Control* (32.9%). The four-class model provided good classification quality, with an entropy value of 0.87 and high average posterior probabilities of class membership: 0.98, 0.93, 0.89, and 0.91, respectively. Figure 2 illustrates the profiles for the four-class solution*.* To aid interpretation, before graphing the profiles, we standardized the scores. For all eight variables included in the LPA, there were significant differences across classes (Kruskal–Wallis *ps* < 0.01). The *High-Complex Challenges* showed impairment on almost all measures, but particularly on emotional lability and irritability. Challenges are also present in PS, high risk-taking, poor self-control, and low future orientation. The one area in which they displayed less impairment was on a self-rating for oversensitivity to immediate rewards (i.e., present hedonism). The *Moderate-Mixed Challenges* class is characterized by strong negative emotionality but to a less intense degree than the *High-Complex class*. This class also still displays elevated issues with self-control, including being drawn to more immediate rewards, yet somewhat higher future orientation and less risk-taking. This *Moderate-Mixe*d class may represent a combination of moderate challenges in cognitive control and reward-related processes. The *Non-Emotive Impulsivity* class is consistent with immature development of cognitive control, appearing highly drawn toward immediate rewards, high risk-taking, low future orientation, and yet, strong emotional control. The *High Regulation and Control* class excelled in cognitive control, reward, and emotional control measures.

**Table 1:** Model fit statistics and estimated class proportions for latent profile models with one to six classes

**Number of classes**	**AIC**	**BIC**	**sBIC**	**CAIC**	**AICc**	**AWE**	**LMRT**	**BLRT**	**cmP**	**Class proportion based on the estimated model**^**a**^					
										**1**	**2**	**3**	**4**	**5**	**6**
One	4817	4865	4814	4881	4821	4993	–	–	< 0.001	1	–	–	–	–	–
Two	4583	4659	4580	4684	4594	4859	< 0.001	< 0.001	< 0.001	0.69	0.31	–	–	–	–
Three	4519	4622	4514	4656	4541	4895	0.13	< 0.001	< 0.001	0.56	0.24	0.19	–	–	–
Four	4471	4602	4465	4645	4508	4947	0.03	< 0.001	0.999	0.33	0.30	0.20	0.17	–	–
Five	4458	4615	4450	4667	4516	5032	0.07	< 0.001	0.001	0.33	0.30	0.20	0.15	0.02	–
Six	4444	4628	4435	4690	4532	5118	0.81	< 0.001	< 0.001	0.30	0.16	0.24	0.14	0.11	0.05

Lower values of AIC, BIC, sBIC, CAIC, AICc, and AWE indicate better model fit. Small *p*-values of the LMRT and BLRT tests indicate that the model with *k* + 1 classes fits the data better than the model with *k* classes. (Vo-Lo-Mendell-Rubin Likelihood Ratio Test *p*-values were also calculated, but they were similar to LMRT and thus were not included in the table.) cmP allows relative comparisons of each of the models to an entire set of 6 models under consideration, providing an estimate of each model being “correct” out of all models considered; the model with the largest value is selected

*AIC *Akaike Information Criterion, *BIC *Bayesian Information Criterion, *sBIC *Sample Adjusted BIC, *CAIC *Consistent AIC, *AICc* Corrected AIC, *AWE *Approximate Weight of Evidence Criterion, *LMRT *Lo-Mendell-Rubin Adjusted Likelihood Ratio Test, *BLRT* Parametric Bootstrapped Likelihood Ratio Test, *cmP *Correct Model Probability

^a^Because of rounding, proportions may not sum to 1

### Differences Among LPA Groups

Demographic and clinical characteristics for all classes are presented in Table 2. The overwhelming majority of the *High-Complex Challenges* class comprises ADHD-diagnosed adolescents*.* The *Moderate-Mixed* class predominantly includes adolescents diagnosed with ADHD, with somewhat lower scores on Conners’ scales than the *High-Complex Challenges class.* The *Non-Emotive Impulsivity* class comprises 59% ADHD-diagnosed adolescents, 2% subthreshold ADHD, and, interestingly, 39% TD. Finally, the *High Regulation and Control* class is primarily, but not exclusively, TD (82%).

**Table 2:** Participant characteristics for the LPA-derived classes

	**High-Complex Challenges** **(*n* = 25)**	**Moderate-Mixed Challenges** **(*n* = 30)**	**Non-Emotive Impulsivity** **(*n* = 46)**	**High Regulation and Control** **(*n* = 51)**	***P-*value**^a^
**Female Gender**, *n* (%)	8 (32%)	8 (27%)	9 (20%)	28 (55%)	0.03
**Diagnosis**, *n* (%)					< 0.001
ADHD	24 (96%)	26 (87%)	27 (59%)	6 (12%)	
Subthreshold ADHD	1 (4%)	1 (3%)	1 (2%)	3 (6%)	
TD	0 (0%)	3 (10%)	18 (39%)	42 (82%)	
**Non-White Race**^**b**^, *n* (%)	4 (16%)	3 (10%)	6 (13%)	1 (2%)	0.27
**Hispanic Ethnicity**^**c**^, *n* (%)	3 (13%)	7 (24%)	6 (14%)	13 (25%)	0.52
**Highest Household Education**^**b**^, *n* (%)					0.98
Less than a bachelor’s degree	5 (20%)	6 (20%)	11 (24%)	10 (20%)	
Bachelor's degree	11 (44%)	11 (36%)	14 (30%)	16 (32%)	
Master's degree	6 (24%)	6 (20%)	10 (22%)	12 (24%)	
Professional/Doctoral degree	3 (12%)	7 (23%)	11 (24%)	12 (24%)	
**Age (years)**, *mean* (*SD*)	14.4 (1.6)	14.2 (1.8)	14.3 (1.7)	14.7 (1.4)	0.43

*LPA *Latent Profile Analysis, *ADHD *Attention-Deficit/Hyperactivity Disorder, *TD *typically developing, *SD *Standard Deviation

^a^For descriptive purposes, participants were assigned to LPA classes using their highest posterior probability. However, analyses accounted for the uncertainty in class assignments. Using 100 pseudo-class draws, we randomly classify youth into latent classes 100 times based on their distribution of posterior probabilities from the best-fitting LPA model and evaluated overall group differences (using *χ*^*2*^ tests for categorical variables and one-way ANOVA for age) 100 times (i.e., for each draw), combined results across draws using standard methods for multiple imputations for missing data. The reported *p*-values reflect these analyses

Data missing for: ^b^*n* = 1; ^c^*n* = 5

Participant sex significantly differed across class membership (*p* = 0.03). A higher proportion of girls were classified in *High Regulation and Control* classes. However, the four classes were similar in age, race and ethnical composition, and parental education (Table 2).

### Differences in Functional Outcomes

**Fig. 3:**
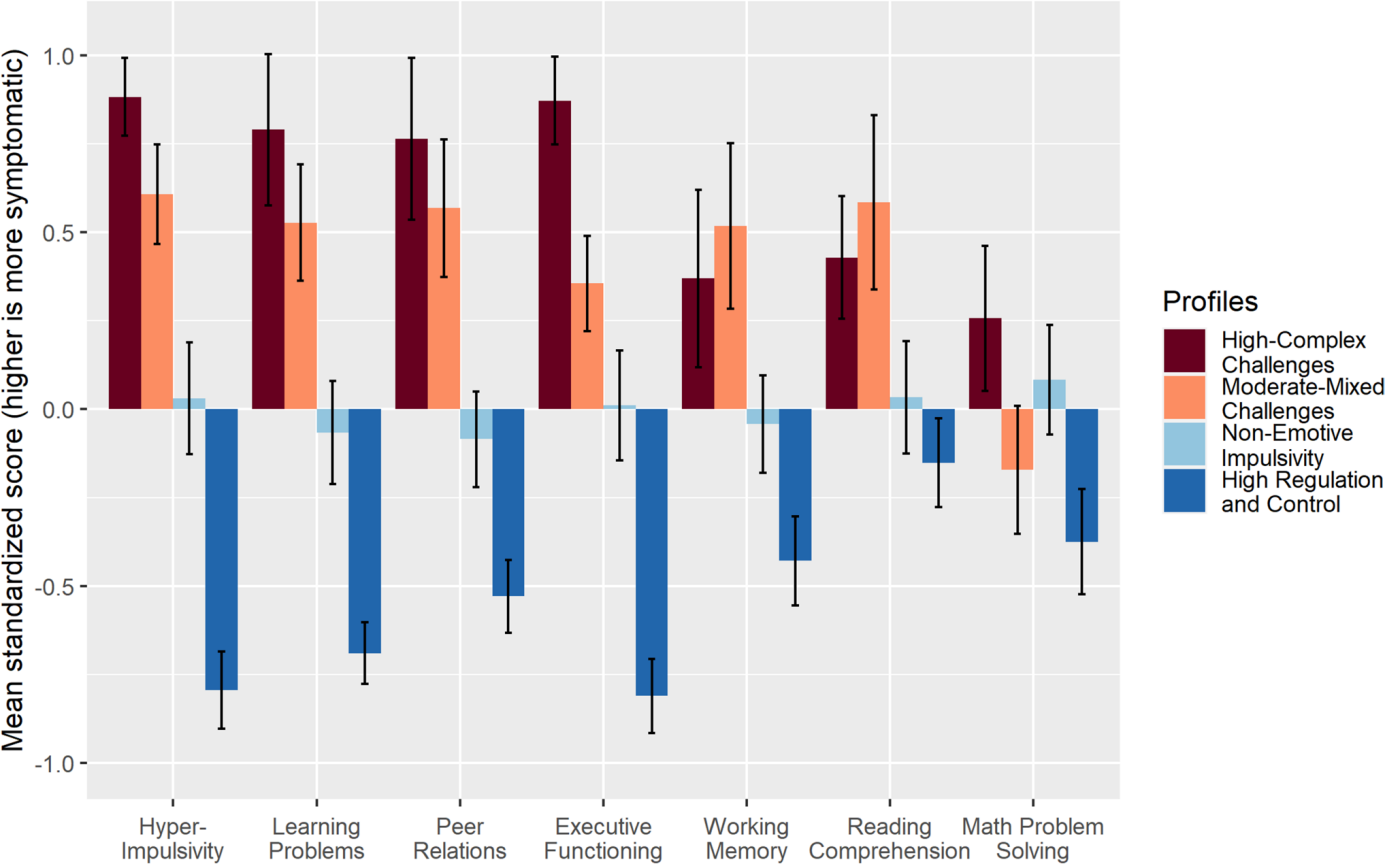
Standardized mean scores for the LPA-derived classes. Standardized mean scores were calculated after rescaling every measure so that higher scores indicate more symptoms. Averages and standard errors for each subgroup were calculated after generating 100 data sets using pseudo-draws to assign group membership and pooling the results. Error bars represent ± 1 standard error

Next, we examined whether the four LPA classes differed in functional outcomes. Table 3 shows the CPRS-3, POMP, and WIAT scores for the four classes, and Fig. 3 summarizes the standardized average scores for the four groups across these variables. Table 4 shows the estimated class differences after adjusting for multiple comparisons.

**Table 3:** Functional outcomes for the LPA-derived classes

	**High-Complex Challenges** **(*n* = 25)**	**Moderate-Mixed Challenges** **(*n* = 30)**	**Non-Emotive Impulsivity** **(*n* = 46)**	**High Regulation and Control** **(*n* = 51)**	***P-*value**^a^
**CPRS T Score**^b^, *mean* (*SD*)					
DSM ADHD Hyper Impulsive	83.0 (11.1)	75.9 (14.4)	67.3 (19.8)	48.7 (12.9)	< 0.001
Learning Problems	69.0 (17.0)	66.1 (12.3)	55.4 (14.2)	45.0 (8.1)	< 0.001
Peer Relations	70.0 (20.5)	64.1 (18.0)	55.8 (15.3)	46.8 (11.1)	< 0.001
Executive Functioning	77.7 (10.6)	73.1 (11.4)	63.6 (16.2)	48.6 (11.1)	< 0.001
**Working Memory Correct**^c.d^, mean (*SD*)	10.2 (3.7)	9.5 (3.5)	11.5 (2.4)	12.7 (2.7)	0.004
**WIAT Standardized Score**^e,f^, mean (*SD*)					
Reading Comprehension	101.9 (11.7)	110.4 (17.2)	107.5 (14.3)	110.3 (11.4)	0.04
Math Problem Solving	105.7 (15.5)	103.9 (13.7)	107.8 (14.7)	114.7 (14.2)	0.04

*LPA *Latent Profile Analysis, *CPRS *Conners’ Parent Rating Scale, *SD *Standard Deviation, *ADHD *Attention-Deficit/Hyperactivity Disorder, *WIAT *Wechsler Individual Achievement Test

^a^For descriptive purposes, participants were assigned to LPA groups using their highest posterior probability. However, to account for the uncertainty in class assignments in analysis, we used 100 pseudo-class draws to randomly classify youth into latent classes 100 times based on their distribution of posterior probabilities from the best fitting LPA model, performed the analyses for overall group differences (general linear models, controlling for sex for DSM ADHD Hyper Impulsive, age and sex for working memory correct, and age in the reading comprehension model) 100 times (i.e., for each draw), combined results across draws using standard methods for multiple imputations for missing data. The reported *p*-values are for *F*-tests from these analyses after further adjusting for multiple comparisons using Benjamini–Hochberg method

Data missing for: ^b^*n* = 1; ^c^*n* = 17; ^e^*n* = 3

^d^This variable was squared-transformed for analysis

^f^These variables were log-transformed for analysis

**Table 4:** Estimated pairwise group differences and 95% confidence intervals for the LPA-derived classes^**a**^

	**High-Complex vs** **Moderate- Mixed**	**High-Complex vs. Non-Emotive Impulsivity**	**High-Complex vs. High Regulation and Control**	**Moderate- Mixed vs. Non-Emotive Impulsivity**	**Moderate Mixed vs. High Regulation and Control**	**Non-Emotive Impulsivity vs** **High Regulation and Control**
**CPRS-3 T Score**^b^						
DSM ADHD Hyper Impulsive	7.5 [-0.9 to 15.8]	17.2 [9.4 to 25.0]^***^	33.2 [25.5 to 40.9]^***^	9.7 [2.0 to 17.5]^*^	25.7 [18.1 to 33.3]^***^	16.0 [8.6 to 23.4]^***^
Learning Problems	3.5 [-3.7 to 10.7]	13.5 [7.0 to 19.9]^***^	23.2 [16.9 to 29.5]^***^	10.0 [3.5 to 16.4]^**^	19.8 [13.4 to 26.1]^***^	9.8 [4.1 to 15.5]^**^
Peer Relations	7.2 [-1.5 to 16.0]	15.0 [7.2 to 22.9]^***^	22.9 [15.2 to 30.6]^***^	7.8 [0.1 to 15.5]	15.7 [8.2 to 23.1]^***^	7.9 [-1.0 to 14.7]
Executive Functioning	4.9 [-2.2 to 12.0]	14.6 [8.0 to 21.2]^***^	28.6 [22.2 to 34.9]^***^	9.7 [3.2 to 16.3]^**^	23.7 [17.5 to 29.9]^***^	13.9 [8.0 to 19.9]^***^
**Working Memory Correct**^c,d^	5.7 [-29.2 to 40.6]	-24.3 [-54.0 to 5.4]	-46.5 [-76.0 to -17.0]^*^	-30.1 [-60.9 to 0.7]	-52.2 [-82.4 to -22.0]^**^	-22.2 [-49.3 to 5.0]
**WIAT Standardized Score**^e,f^						
Reading Comprehension	-0.07 [-0.13 to -0.001]	-0.05 [-0.11 to 0.01]	-0.08 [-0.14 to -0.02]^*^	0.02 [-0.04 to 0.08]	-0.01 [-0.07 to 0.04]	-0.03 [-0.08 to 0.02]
Math Problem Solving	0.001 [-0.07 to 0.08]	-0.02 [-0.09 to 0.04]	-0.09 [-0.15 to -0.02]	-0.03 [-0.09 to 0.04]	-0.09 [-0.15 to -0.02]	-0.06 [-0.12 to -0.001]

*LPA *Latent Profile Analysis, *CPRS *Conners’ Parent Rating Scale, 3^rd^ Ed., *ADHD *Attention-Deficit/Hyperactivity Disorder, *WIAT *Wechsler Individual Achievement Test

^*^p < 0.05; ^**^p < 0.01; ^***^p < 0.001, for pairwise group comparisons, after adjusting for multiple comparisons for each variable

^a^Data reported are estimated difference [95% confidence intervals]. General linear models were used to assess group differences, controlling for sex for DSM ADHD Hyper Impulsive, age and sex for working memory correct, and age in the reading comprehension model. To account for the uncertainty in class assignments in analysis, we used 100 pseudo-class draws to randomly classify children into latent classes 100 times based on their distribution of posterior probabilities from the best fitting LPA model, performed the analyses 100 times (i.e., for each draw), and combined results across draws using standard methods for multiple imputations for missing data

^d^This variable was squared-transformed for analysis

^f^These variables were log-transformed for analysis

Data missing for: ^b^*n* = 1; ^c^*n* = 17; ^e^*n* = 3

The classes differed significantly on all CPRS scales examined (all *p*< 0.001). After adjusting for multiple comparisons, adolescents in the *High-Complex* class displayed significantly higher DSM ADHD Hyperactive/Impulsive scores compared to two classes, *Non-Emotive Impulsivity* and *High Regulation and Control* (*p*< 0.001; Table 4). The *Moderate-Mixed* class had significantly higher scores than the *Non-Emotive Impulsivity* and *High Regulation and Control* classes (*p* = 0.03 and < 0.001, respectively). After adjusting for multiple comparisons, the *High-Complex* class did not significantly differ from the *Moderate-Mixed* class (*p* = 0.08). Finally, the *Non-Emotive Impulsivity* class had significantly higher scores than the *High Regulation* and *Control* class (*p*< 0.001). A similar pattern was observed for Learning Problems, with the *High-Complex* class scoring higher than the *Non-Emotive Impulsivity* and *High Regulation and Control* (both* p* < 0.001)*.* The *Moderate-Mixed* class showed more Learning Problems than the *Non-Emotive Impulsivity* and the *High Regulation and Control* classes (*p* = 0.005 and < 0.001, respectively). Finally, the *Non-Emotive Impulsivity* class had higher scores on Learning Problems than the *High Regulation and Control* class (*p* = 0.002). Peer Relations from the CPRS-3 revealed significant differences with the *Moderate-Mixed* class, indicating higher Peer Relations scores than the *High Regulation and Control* class (*p* < 0.001). Additionally, the *Non-Emotive Impulsivity* class had higher Peer Relations scores than the *High Regulation and Control* class, but this difference was not statistically significant (*p* = 0.07). Finally, the *High-Complex* class had higher scores on the Peer Relations subscale compared to the *Non-Emotive Impulsivity* and *High Regulation and Control* classes (both *p*< 0.001). The pattern was similar for EF, with the *High-Complex* class scoring higher than the *Non-Emotive Impulsivity* and *High Regulation and Control* classes (*p*< 0.001). Additionally, the *Moderate-Mixed* class scored higher on EF than the *Non-Emotive Impulsivity* and *High Regulation and Control* classes (*p* = 0.007 and < 0.001, respectively). The *Non-Emotive Impulsivity* class scored higher on EF than the *High Regulation and Control* class (*p* < 0.001).

The groups differed significantly on the squared transformed WM task scores (*p* = 0.004). After further adjusting for multiple comparisons, the results showed that adolescents in the *High-Complex* and *Moderate-Mixed* classes had lower scores compared to the *High Regulation and Control* class (*p* = 0.01 and 0.004, respectively). Finally, we examined whether the LPA classes differed in academic achievement based on WIAT-III standardized Reading Comprehension and Math Problem Solving scores. As expected, the classes differed on the log-transformed WIAT-III standardized subscale scores (both *p* = 0.04 after adjusting for multiple comparisons). After further adjusting for multiple comparisons, the results indicated that adolescents in the *High-Complex* class performed significantly worse in Reading Comprehension than those in the *High Regulation and Control* class (*p* = 0.03, Table 4). For the Math Problem Solving scores, the results suggested that adolescents in the *High-Complex class* demonstrated worse performance than those in the *High-Regulation and Control* class in Math Problem-solving. Still, these differences did not reach statistical significance after adjusting for multiple comparisons (both *p* = 0.055, Table 4).

## Discussion

This study used a dimensional approach to identify classes of performance regarding “hot” and “cold” functioning in adolescents who were primarily TD or diagnosed with ADHD Combined Presentation and to characterize further whether classes differed regarding emotionality, irritability, impulsiveness, time perspective, cognition, and risk-taking. Our results revealed four classes with varying ADHD characteristics. The *High-Complex* class is composed of females (32%) and males (68%) with ADHD (96%) and subthreshold ADHD (4%). The literature on male-to-female prevalence for ADHD, including for Combined Presentation, which comprises most of the ADHD group, reports a wide range for the male-to-female ratio, and the percentage of females in this class reflects the higher end of that range (Danielson et al., 2018; Willcutt et al., 2012). The relatively high percentage of females in this class is consistent with other findings (e.g., Rosch et al., 2018), where females may exhibit more severe ADHD symptoms, particularly regarding impulsivity. This group displays high levels of hot and cool processing challenges, potentially associated with overactive ventral striatum and amygdala activity and connectivity and reduced activity in cortical brain regions and connectivity between cortical and subcortical regions.

The *Moderate-Mixed* class comprises predominantly male individuals (73%) with a range of symptom severity, including moderate levels of lability and irritability. However, difficulties with self-control were also evident. Additionally, this class displayed somewhat lower risk-taking behaviors. Weaknesses in future orientation may weaken their self-control. We hypothesize that adolescents in this class may eventually outgrow some of their challenges and are less likely to have high comorbid disorders than those in the *High-Complex* class*.* The *Non-Emotive Impulsivity* class primarily comprises males (80%). This class displayed lower levels of emotionality with reduced irritability, yet difficulties with self-control, low future orientation, and high risk-taking. Their profile is somewhat consistent with a profile of younger youth, perhaps with under-developed EF and heightened responsivity to immediate rewards. Their PS was weaker, which could predict potential difficulties with tasks that require executive control (Barkley, 2013; Wilcutt et al., 2005).

We hypothesize that longitudinal data of these adolescents may find that those in this group who are diagnosed with ADHD may have a remittance of symptoms as they mature. Findings from structural imaging studies (Lenroot & Giedd, 2010) suggest males are about two to three years slower in brain development than girls. We speculate that parents of a subset of this class who have concomitant lower grades and poor study habits may experience concern when their children reach high school. Specifically, parents may worry about their adolescents' maturity level and ability to compete and prepare for college, motivating them to seek an ADHD evaluation and treatment. Finally, the *High Regulation and Control* class is characterized by a relatively even distribution of males (45%) and females (55%), with most individuals presenting with TD profiles (82%). Overall, individuals in this class performed uniformly well across modalities but demonstrated particularly strong EF, self-control, and future orientation. However, approximately 18% of individuals within this class were diagnosed with either ADHD or sub-threshold ADHD, indicating that there may be varying degrees of core symptom manifestation. Thus, these youth are exhibiting behaviors that are sufficiently severe to warrant a diagnosis (or sub-threshold diagnosis) of ADHD. Yet, in the behaviors we examined in this analysis they were more similar to TD peers. Other studies have found that only 33% to 50% of children diagnosed with ADHD also present with executive dysfunction (Kofler et al., 2019), and only 20% to 50% of children diagnosed with ADHD experience irritability and have difficulty with emotional regulation (Shaw et al., 2014). Consequently, these findings suggest that among the 18% of individuals with ADHD in the class, it is plausible that they may not exhibit these symptoms to the same extent as other children with ADHD. They may have different strengths that allow them to compensate on a neural and/or behavioral level to control their emotions and impulsive behavior. Our findings reinforce the concept that there are important subgroups within the broad diagnostic category of “ADHD diagnosis,” even within the ADHD Combined Presentation, presenting with profiles that vary according to different strengths and weaknesses.

Our second objective was to investigate whether there were any differences between ADHD classes in terms of functional outcomes. A review of the relation between the classes and the functional variables revealed that the *High-Complex* class demonstrated uniformly high challenges in hyperactive/impulsive and EF behaviors and school-related issues, including Peer Relations. The *Moderate-Mixed Class* showed less intense emotional challenges than the *High Complex-Class*, a relative strength in Math Problem Solving, yet greater WM and Reading Comprehension challenges. These findings suggest a disassociation between the type of academic challenge (i.e., math versus reading) and perhaps their relation to WM performance.

Our WM task is an object span task rather than a verbal task, and it may be that a verbal WM task would have produced different results. The *Non-Emotive Impulsivity* class demonstrated significant differences in the Conners’ functional measures (i.e., Hyperactivity/ Impulsivity, Learning Problems, Peer Relations and EF) compared to the other classes. Yet, after controlling for multiple comparisons, their academic performance appears to be relatively intact, in comparison to the *High Regulation and Control* class. Future research should explore if there are different neural mechanisms associated with the degree of emotionality expressed, WM, and academic performance between the classes.

We suspect persons in the *High-Complex* class will likely experience the most persistent challenges. For example, Barkley and colleagues (2008) found greater emotional impulsivity and lability (frustration, annoyance, anger) in a prospective study of adults who were diagnosed with ADHD as children when their ADHD persisted to 27 years of age in comparison to adults whose childhood ADHD did not persist into their late 20s. Furthermore, work in the Hinshaw laboratory found that girls with ADHD with greater impulsivity were more likely as young adults to exhibit self-harm: suicidal ideation and attempts and serious non-suicidal severe injury (Hinshaw et al., 2012). Thus, we recommend that parents and practitioners continue to closely monitor and provide treatment for adolescents in this class, as future challenges are likely to emerge. It will be interesting to see if youth in the *Non-Emotive Impulsivity class* either outgrow their symptoms or learn to manage them to minimize their negative impact on other aspects of their lives.

Adolescence is a time of significant developmental changes, both biologically and socially, which can impact the presentation of ADHD symptoms. Although these individuals were selected for high ADHD hyperactive/impulsive symptoms, these symptoms tend to decline during adolescence and adulthood for some, while others continue to experience symptoms into adulthood (Barkley, 2015). This study highlights the importance of understanding how ADHD presents during different developmental periods, including adolescence, and the potential limitations of relying on a categorical diagnostic system like the DSM.

Our findings provide hints as to which aspects of functioning are more amenable to prevention and intervention. Recent research (Brotman et al., 2017) is testing ways to target irritability, which may improve long-term outcomes for those youth presenting with high irritability and emotional lability. Interventions to increase the delay of gratification in young children by using shaping techniques to increase patience while waiting for delayed rewards or reinforcing the use of alternative rewards while waiting for delayed rewards (e.g., Schweitzer & Sulzer-Azaroff, 1988, 1995) may be helpful for many children expressing high impulsivity and “present hedonism.” Episodic future thinking might be particularly useful for those adolescents who have weak future orientation (e.g., *High-Complex* and *Moderate-Mixed* classes and has received surprisingly little attention in the ADHD research community (c.f., Solanto & Scheres, 2021) but has strong empirical support for improving self-control in youth (Daniel et al., 2015) and adults struggling with addiction (Bickel et al., 2015). Similarly, adolescents in three of the four classes might have benefitted when they were younger from work from Diamond and colleagues from the “Tools of the Mind” curriculum, which targets teaching self-control in a naturalistic classroom setting (Diamond et al., 2019). Cognitive training programs, which tend to target WM or attention, may perhaps be more effective in enhancing functioning in youth who display weak PS (Schiff et al., 2021; Sonuga-Barke et al., 2014), enabling them to more rapidly perceive and act on information to guide their general behavior. It remains to be seen if targeting these narrower cognitive processes will generalize to more broad functions, including improving emotional reactivity and self-control when youth encounter seductive, immediately available rewards or are in intensely emotional situations. Researchers should also consider possible moderators and mediators to help develop personalized prevention and intervention approaches that consider biological factors (e.g., sex, hormones) and state factors (e.g., stress, sleep, type of reward [social, non-social, food, monetary]) when developing approaches for ADHD.

Future research directions include examining how these classes relate to brain function and structure. This includes more research on sex differences in relation to distinguishing between the different profiles. Although ADHD is more commonly diagnosed in males than females, recent research has suggested that females with ADHD may present differently than males, with potentially different patterns of symptoms, comorbidities, and functional impairments (Danielson et al., 2018; Skogli et al., 2013; Willcutt et al., 2012). More research on sex differences in ADHD could help refine diagnostic criteria and treatment approaches for both males and females with ADHD. Finally, exploring if these same classes are evident in adult samples will be valuable.

## Limitations

This study had a relatively low sample size for a latent class approach; therefore, we had limited power to detect smaller subgroups or groups that were not well separated. This limitation impacts the generalizability of our findings to children and adolescents with ADHD. Larger studies with adequate power are needed to identify subtypes reliably. However, findings from studies with smaller sample sizes, such as ours, can still be informative and function as a foundation to guide future hypotheses. Studies with modest sample sizes often benefit from a more comprehensive, thorough characterization with multiple measures. They can also serve as a launching pad to explore more intricate phenotypes with greater depth in larger data sets. Many of the larger data sets available lack the range of measures that this project encompasses, thereby providing a richer and more comprehensive understanding of ADHD. The insights gained from this study not only contribute to the refinement of research questions but also offer valuable guidance for selecting future measures, ensuring a more nuanced investigation of ADHD symptoms. A consideration with our sample is that youth meeting the criteria for diagnoses of depression or major anxiety disorders (i.e., not phobias) were excluded from the study, thus limiting the generalization of our ADHD sample to the general ADHD population. Many of our youth, however, did endorse depressive or anxious symptoms, though insufficient to meet diagnostic criteria. Finally, while the study measures are widely used in research and clinical practice, we recognize that many are parent or self-report measures (i.e., CPRS-3, ZTPI, BIS) and are subject to shared method variance.

## Conclusion

In summary, our study provides evidence of distinct ADHD symptom classes in relation to emotionality, impulsivity, self-control, risk-taking, and PS in adolescents with and without ADHD. These findings could assist clinicians in identifying youth who display greater differences in ADHD and emotional symptoms, potentially aiding in the development of more effective interventions for difficulties with emotional regulation, attention, hyperactivity, and impulsivity. Our findings suggest a potential rationale for investigating the effectiveness of ADHD interventions personalized to the unique needs of individuals based on their ADHD class.

## Financial Disclosures

Dr. Hinshaw receives book royalties from Guilford Press, Oxford University Press and St. Martin’s Press. Dr. Iosif has received honoraria for reviewing activities from Elsevier. Ms. Elahi, Drs. Mukherjee and Schweitzer report no competing interests.

## Data Availability

Data is available upon request.
